# Aspirin inhibits RANKL-induced osteoclast differentiation in dendritic cells by suppressing NF-κB and NFATc1 activation

**DOI:** 10.1186/s13287-019-1500-x

**Published:** 2019-12-05

**Authors:** Lili Wu, Zhenhua Luo, Yitong Liu, Lu Jia, Yiyang Jiang, Juan Du, Lijia Guo, Yuxing Bai, Yi Liu

**Affiliations:** 1 Laboratory of Tissue Regeneration and Immunology and Department of Periodontics, Beijing Key Laboratory of Tooth Regeneration and Function Reconstruction, School of Stomatology, Capital Medical University, Tian Tan Xi Li No.4, Beijing, 100050 People’s Republic of China; 2 Department of Orthodontics, School of Stomatology, Capital Medical University, Beijing, China

**Keywords:** Aspirin, Dendritic cells, Osteoclasts, Receptor activator for nuclear factor-κB ligand (RANKL)

## Abstract

**Background:**

Aspirin has been demonstrated to promote osteoblast-mediated bone formation and inhibit osteoclast (OC)-mediated bone resorption. However, it remains unclear whether aspirin influences other immune cells during bone resorption. Dendritic cells (DCs), the most potent antigen-presenting cells, can also transdifferentiate into active OCs in the presence of receptor activator of nuclear factor-κB ligand (RANKL) and macrophage colony-stimulating factor (M-CSF). The effects of aspirin on DC-derived OCs (DDOCs) were investigated in the current study.

**Methods:**

Flow cytometry and mixed lymphocyte reaction (MLR) assays were used for DC identification. The proliferative capacity of DCs was determined by BrdU assays. Apoptosis was examined by flow cytometry. The osteoclastic potential of DCs was tested using tartrate-resistant acid phosphatase (TRAP) staining, western blotting, and reverse transcription polymerase chain reaction (RT-PCR). Western blotting was also used to examine signaling pathways. A mandibular bone defect model was established to assess the effect of aspirin on bone resorption.

**Results:**

Aspirin had no influence on the surface phenotype, proliferation, or apoptosis of DCs, though aspirin significantly inhibited osteoclast differentiation in RANKL-stimulated DCs. DC osteoclast differentiation was modulated by aspirin via the nuclear factor kappa B (NF-κB)/nuclear factor of activated T cell, cytoplasmic 1 (NFATc1) signaling pathway. Aspirin treatment also had favorable therapeutic effects on bone regeneration in the bone defect model, and the number of osteoclasts was decreased.

**Conclusions:**

Aspirin inhibited RANKL-induced OC differentiation in DCs via the NF-κB pathway, downregulating expression of NFATc1. Aspirin treatment promoted bone regeneration by inhibiting DDOC activation in the early stages of inflammation in a rat mandibular bone defect model.

## Background

Bone homeostasis is maintained through the balance between the activity of osteoclasts (OCs) and that of osteoblasts. However, in some inflammatory diseases, such as osteoporosis and periodontitis, bone resorption exceeds bone formation, creating an imbalance caused by abnormal activation of OCs [1].

OCs, unique cells capable of dissolving bone matrix, are derived from the monocyte/macrophage lineage of hematopoietic precursors. OC differentiation and activation is regulated by two critical cytokines: macrophage colony-stimulating factor (M-CSF) and receptor activator of nuclear factor kB ligand (RANKL) [2]. During differentiation, the fusion of mononuclear precursors is indispensable for the formation of mature multinucleated OCs, which play an essential role in resorbing bone [3].

In addition to the major involvement of OCs in bone homeostasis, the involvement of other immune cells in bone resorption has been shown in several studies. Dendritic cells (DCs), the major antigen-presenting cells, also share several features with OCs; they are derived from the same myeloid precursor, and the functions of both cell types are closely dependent upon RANKL [4]. Interestingly, there is increasing evidence that immature DCs (imDCs) have osteoclastogenic potential in some inflammatory pathological conditions because imDCs can transdifferentiate into active OCs after stimulation with RANKL and M-CSF [5, 6]. These findings clearly highlight a potential link between DCs and OCs.

Because of the major involvement of OCs in bone homeostasis, a feasible strategy is to inhibit osteoclastogenesis and/or decrease bone resorption activity in active OCs to prevent or treat bone resorption-related disorders. Aspirin is a classic nonsteroidal anti-inflammatory drug (NSAID) that is well known for its antipyretic, analgesic, and anti-inflammatory effects. Aspirin irreversibly inhibits cyclooxygenase-1(COX-1) and cyclooxygenase-2 (COX- 2) activities via covalent modification [7]. Moreover, multiple animal and clinical studies have reported that COX-2 inhibition negatively affects bone healing [8–11], though a small minority of these studies have demonstrated no lasting negative effects [11–13]. Recent studies have shown that aspirin can regulate the bone balance in ovariectomy-induced osteoporosis [7]. Furthermore, aspirin was found to inhibit RANKL-induced osteoclastogenesis in RAW264.7 cells [14]. However, the effects of aspirin on bone healing remain highly debated. The main factors that may underlie the discrepancies between studies are the mode of administration, the variability of the drug dose, the dosing duration, and the type of bone defect model [11]. According to our previous studies, aspirin is able to promote bone marrow mesenchymal stem cell (BMSC)-based skull bone regeneration in mini-pigs after release in a local, sustained, and controlled manner [15]. In the present study, we examined whether aspirin regulates dendritic cell-derived osteoclast (DDOC) formation and activation.

## Materials and methods

### Animals

C57BL/6J mice (male, aged 8–10 weeks) and rats were sourced from the Institute of Vital River (Beijing, China). This study was performed according to institutionally set guidelines for animal research.

### Isolation and culture of CD11c^+^ DCs

Murine DCs were generated from bone marrow precursors. Bone marrow single-cell suspensions were generated from tibias and femurs and filtered through a 70-μm strainer (BD Bioscience, CN). The cells were then incubated with Red Blood Cell Lysis Buffer (APPLYGEN, China) and washed. The cells were then seeded in six-well plates (10^6^ cells/ml) in RPMI-1640 medium (Gibco, USA) containing 10% heat-inactivated fetal bovine serum (FBS), 100 μg/ml streptomycin, 100 U/ml penicillin, and 2 mM l-glutamine and supplemented with 25 ng/mL recombinant mouse GM-CSF (rmGM-CSF; R&D Systems, USA). The cells were induced for 3–7 days, and then imDCs were induced to mature by the addition of TNF-alpha (PeproTech, USA, 20 ng/ml) for 24 h before harvesting on the 7th day.

### Surface markers of DCs after aspirin treatment

DCs cultured with or without aspirin were prepared for surface marker expression analysis by FACS staining. Cells were incubated on ice for 15–30 min with specific monoclonal antibodies (mAbs): FITC-labeled anti-CD11c, PerCP/Cy5.5-labeled anti-MHC class II, PE-labeled anti-CD80, and APC-labeled anti-CD86 (BD Biosciences, USA). After staining, the labeled cells were analyzed with a BD FACSCanto II flow cytometer (BD Biosciences).

### Isolation and culture of CD4^+^ T cells

A mixed lymphocyte reaction (MLR) assay was employed to determine the functional activity of DCs; T lymphocytes were used as responder cells. CD4^+^ T cells were isolated from splenocyte suspensions and sorted by MIDI magnetic cell sorting (MACS; Miltenyi Biotec, Germany). Naive CD4^+^ T cells were incubated in T cell culture medium (RPMI-1640 medium containing 10% FBS, 2 mmol/l-glutamine, 100 μg/ml streptomycin, 100 U/ml penicillin, and 20 mol/L HEPES; Invitrogen, USA). The naive CD4^+^ T cells were activated using soluble anti-mouse CD28 (2.5 μg/ml) and plate-bound anti-mouse CD3 (5 μg/ml) antibodies.

### MLR assay

To analyze T cell proliferation, cells (1 × 10^6^) were labeled with 5 mM carboxyfluorescein succinimidyl ester (CFSE; Life Technologies, USA) for 15 min in PBS (1% BSA). The cells were seeded in round-bottom 96-well plates to ensure effective DC/T cell interaction, after which DCs were added in gradient doses to activate the T cells (1 × 10^5^ cells per well). The cells were cultured for 72 h, and the proliferation of the CD4+ T cells in response to priming by the DCs was assessed by FACS. Data were analyzed using ModFit LT.

### BrdU assay for cell proliferation

ImDC proliferation was evaluated with BrdU Cell Proliferation Assay Kit (BioVision, USA). DCs (5.0 × 10^3^ cells/well) were seeded in triplicate in a 96-well flat-bottom plate (Costar, USA) and incubated in 200 μl of standard culture medium with or without aspirin for 24 h. The cultures were then incubated with a BrdU solution for 4 h. Absorbance at 450 nm was measured using an enzyme-linked immunosorbent assay (ELISA) reader (ELx800; Bio-Tek Instruments Inc., USA), with the optical density (OD) value at 450 nm representing proliferating cells.

### Apoptosis assay

To detect apoptotic cells in the presence or absence of aspirin, apoptosis was assessed using Annexin V Apoptosis Detection Kit FITC (eBioscience, USA).

### OC differentiation and the tartrate-resistant acid phosphate (TRAP) assay

imDCs were further differentiated with α-minimum essential medium (α-MEM; Gibco, USA) containing 100 ng/ml RANKL and 25 ng/ml M-CSF to obtain OCs.

A leukocyte acid phosphatase kit (Sigma-Aldrich) was utilized to examine the TRAP activity of adherent OC cultures. If TRAP-positive cells had three or more nuclei, they were considered OCs. TRAP-positive cells were analyzed by cell counting in at least five random fields under a light microscope (OLYMPUS, Japan).

### Reverse transcription polymerase chain reaction (RT-PCR)

imDCs (10^6^ cells/ml) were plated in a six-well plate with various concentrations of aspirin and cultured for 5–7 days in culture medium. Total RNA was isolated from the cultured cells with TRIzol reagent (Invitrogen, USA) and reverse transcribed into cDNA using oligo (dT) according to the manufacturer’s protocol (Invitrogen, USA). Real-time PCR was performed using iCycler iQ Multi-Color Real-time PCR Detection System and a QuantiTect SYBR Green PCR kit (Qiagen, Germany). The specific primer sequences used are listed in Table 1.

**Table 1:** Primers used for RT-PCR

Gene symbol	Sequence(5′—>3′)
Mouse TRAP	(F)AGCAGCCAAGGAGGACTAC
	(R)CATAGCCCACACCGTTCTC
Mouse CTSK	(F)AGCTTCCCCAAGATGTGAT
	(R)AGCACCAACGAGAGGAGAA
Mouse CTR	(F)CTCCTTGTCGATTGCTGCT
	(R)TCACCCTCTGGCAGCTAAG
Mouse GAPDH	(F) TGTAGACCATGTAGTTGAGGTCA
	(R) AGGTCGGTGTGAACGGATTTG

*F* forward, *R* reverse

### Western blot analysis

imDCs were plated in six-well plates and treated with aspirin for 24 h prior to treatment with RANKL and M-CSF. The cells were lysed in RIPA Lysis Buffer (Beyotime, China). Nuclear proteins were extracted using an EpiQuik Nuclear Extraction kit (EpiGentek, USA). Protein extracts were separated by 10% SDS polyacrylamide gel electrophoresis (Applygen, China), and subsequent processes were performed following a standard protocol.

### Mandibular bone defect model

We established a mandibular bone defect in rats ranging from the incisors to the molars, 5 mm long, 2 mm wide, and 1 mm high, under general anesthesia (10% pentobarbital, 40 mg/kg). After surgery, all rats were randomly divided into aspirin and control groups. We mixed hydrogel (500 μl, BeaverBio, China) and hydroxyapatite/tricalcium phosphate (HA/TCP) (20 mg) with or without 150 μg/ml aspirin and filled the mandibular bone defect with this mixture, followed by suturing the incisions intermittently with 0–4 absorbable sutures (Fig. 5a). The rats were sacrificed on days 3 and 14 and at 2 months.

### Histological evaluations of the bone defect

After rats were sacrificed, the defective area in the rat mandibular bone was observed by stereomicroscopy. After the mandibular bones were fixed in 4% paraformaldehyde (PFA), they were decalcified with buffered 10% EDTA (pH 8.0) and embedded in paraffin. The tissues were sectioned at 5 μm and stained with TRAP and hematoxylin and eosin (H&E).

### Immunohistochemical staining

We euthanized rats after surgery to perform immunohistochemical examinations. Paraffin-embedded sections acquired as described above were dewaxed in xylene and dehydrated in alcohol. To reduce nonspecific staining, all sections were incubated with 3% hydrogen peroxide and then blocked with 10% serum. Next, the samples were incubated with a mouse anti-rat CD11c antibody (1200, Abcam, UK) and treated with biotinylated secondary antibodies. After the samples were developed by DAB staining and counterstained, observations were performed with a confocal microscope (OLYMPUS, Japan). Positively stained cells were counted in at least five random fields and quantified using Image-Pro Plus 6.0.

### Statistical analysis

GraphPad Prism7 was used for statistical analysis. Unless otherwise noted, statistical significance comparisons of two groups were performed by Student’s *t* test, and one-way ANOVA was applied for comparisons among multiple groups. For samples with heteroscedasticity, Kruskal-Wallis and Mann-Whitney *U* tests were used to evaluate differences.

## Results

### Isolation and characterization of DCs

**Fig. 1:**
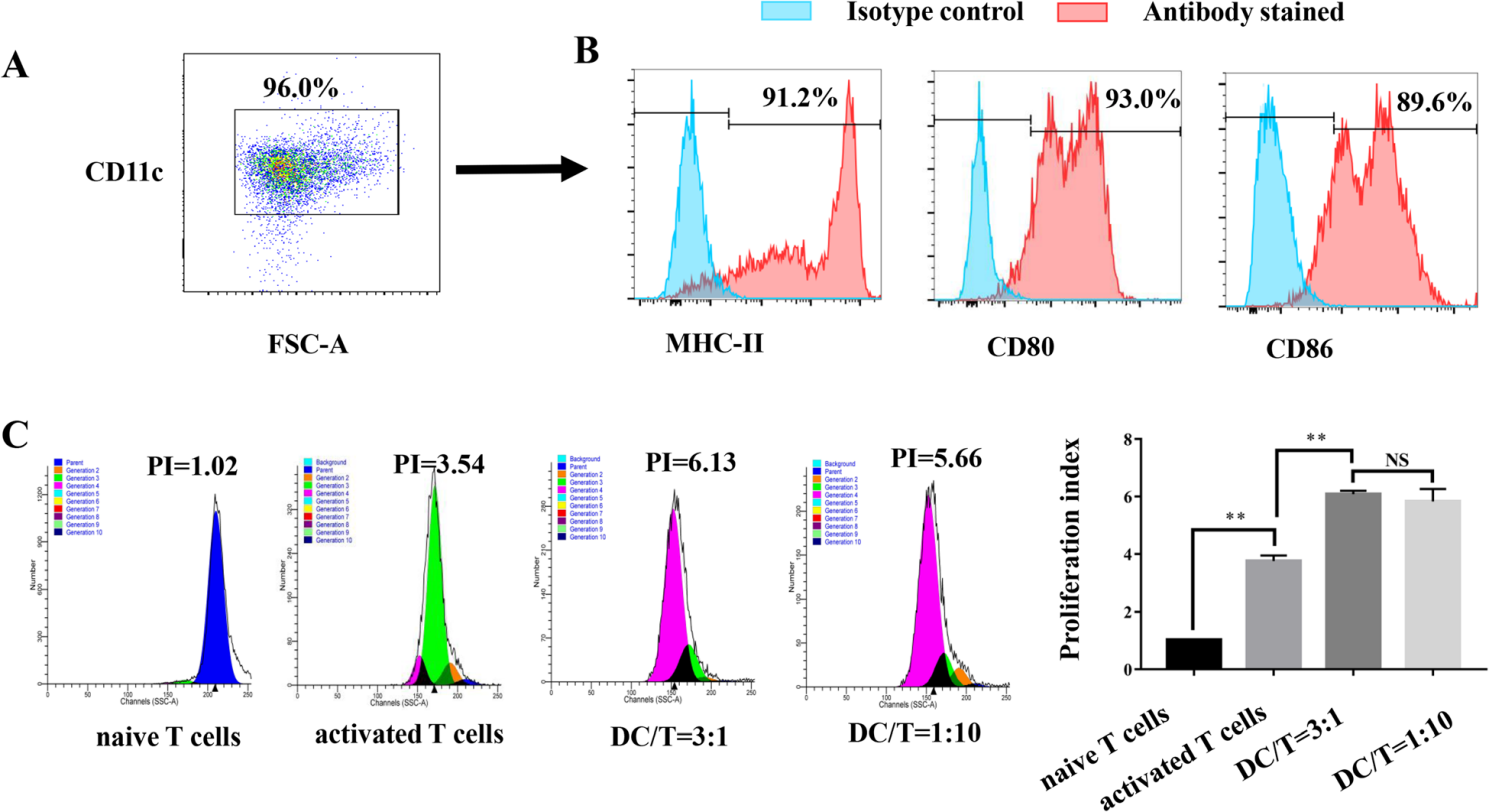
Dendritic cells (DCs) have greater T cell proliferation promotion ability. **a**, **b** DCs generated from wild-type mice expressed high levels of CD11c, MHC class II, CD80, and CD86 surface molecules. **c** DCs induced proliferation of allogeneic CD4^+^ T cells in a mixed lymphocyte reaction (*P* < 0.01). No significant differences were detected in the levels of T cell proliferation when different ratios of DC/T cells were cultured and harvested (*P* > 0.05). All experiments are representative of three replicates. *P < 0.05, **P < 0.01

imDCs can be stimulated to mature by exposure to cytokines. TNF-α induced maturation of DCs, which was characterized by significantly increased expression of CD11c, the costimulatory molecules CD80 and CD86, and the antigen-presenting molecule MHC class II (Fig. 1a, b).

The DCs were then tested for their ability to promote the proliferation of allogeneic CD4^+^ T cells in an MLR assay. CFSE-labeled naive CD4^+^ T cells were cocultured with different numbers of DCs. The cells were harvested on day 3, and the number of proliferating cells had increased (Fig. 1c; *P* < 0.01).

### Effect of aspirin on imDC proliferation and apoptosis

**Fig. 2:**
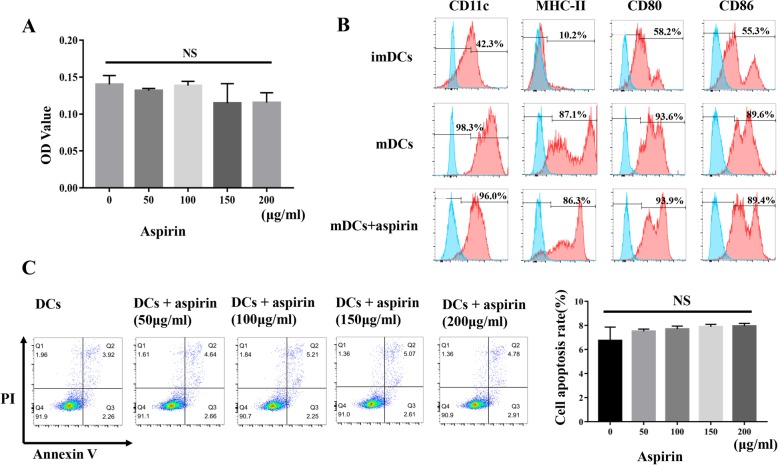
Aspirin has no influence on DC apoptosis, proliferation, or immunophenotype. **a** Aspirin was not found to exert different effects on cell proliferation at the concentrations tested (*P* > 0.05). **b** Immature DCs (imDCs) expressed low levels of CD11c, MHC class II, CD80, and CD86 surface molecules; mature DCs highly expressed these surface molecules. Expression of surface markers on DCs with or without aspirin treatment showed no obvious differences. **c** The addition of aspirin had no influence on DC apoptosis (*P* > 0.05). All experiments are representative of three replicates. *P < 0.05, **P < 0.01

The effects of aspirin on imDCs were evaluated by culturing imDCs with aspirin at different concentrations, followed by assessment of the rates of cell proliferation and apoptosis. Aspirin did not have different effects on cell proliferation at the concentrations tested (Fig. 2a; *P* > 0.05). Additionally, aspirin did not dramatically induce apoptosis in imDCs (Fig. 2c; *P* > 0.05).

### Aspirin was inefficient at modulating the DC surface phenotype

Cells were treated with aspirin overnight before maturation with TNF-α. The aspirin-treated DCs displayed a surface expression pattern similar to that of untreated cells, as the levels of CD11c, CD80, CD86, and MHC-II expression on the cell surface were higher than those on the imDC cell surface (Fig. 2b).

### Aspirin inhibited OC differentiation in RANKL-stimulated imDCs

**Fig. 3:**
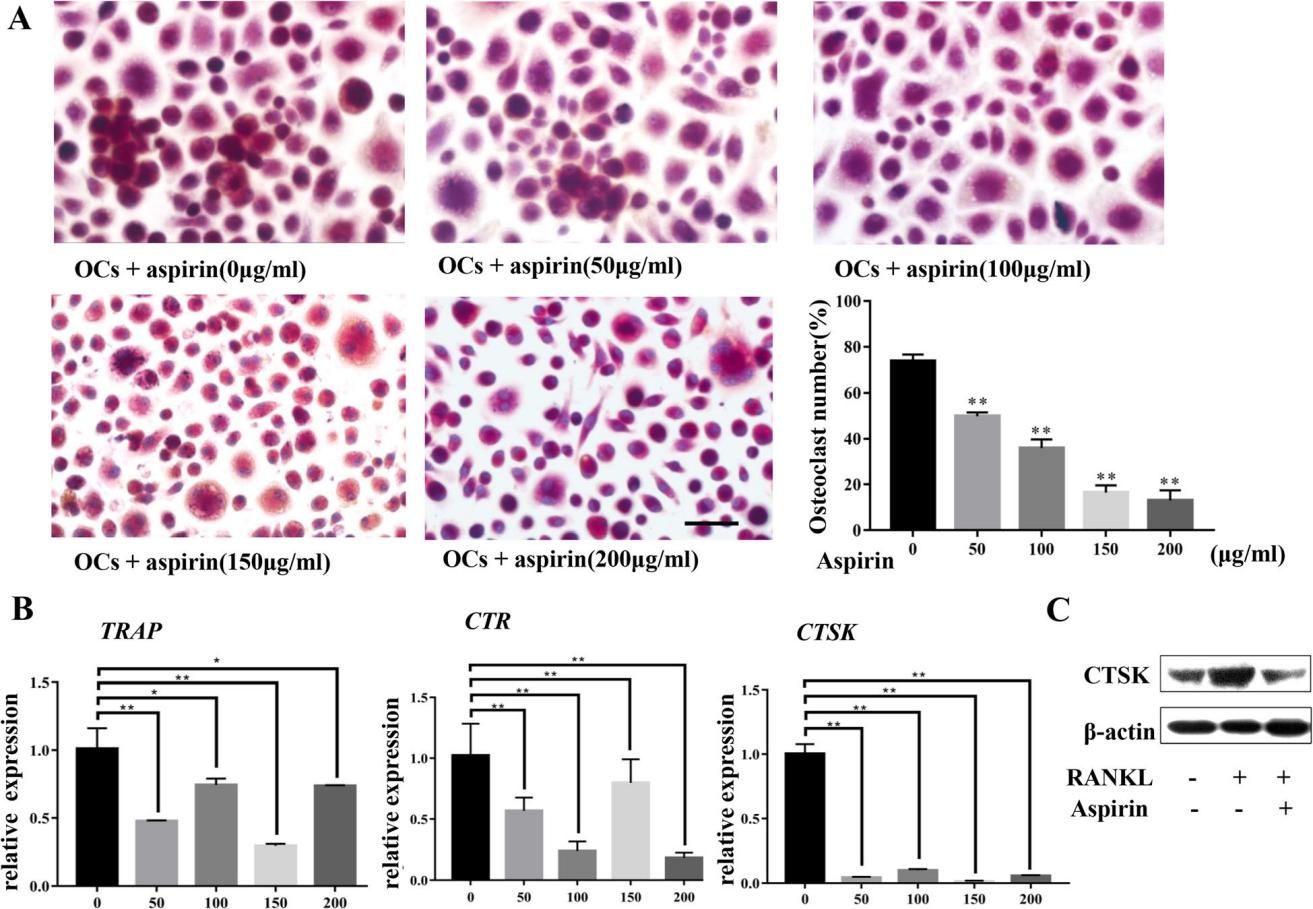
Aspirin inhibits osteoclast differentiation in RANKL-stimulated imDCs. **a** TRAP^+^ multinuclear cells (MNCs) with more than three nuclei were considered mature osteoclasts (OCs). TRAP staining showed that aspirin significantly suppressed the formation of RANKL-induced osteoclast-like cells among imDCs (*P* < 0.01). **b** RT-PCR showed that aspirin treatment downregulated expression of tartrate-resistant acid phosphatase (TRAP), calcitonin receptor (CTR), and cathepsin K (CTSK) (*P* < 0.01). **c** Consistent with the RT-PCR results, western blot analysis showed that CTSK expression levels were increased during osteopetrosis induction but that aspirin treatment partially suppressed this increase. All experiments are representative of three replicates. *P < 0.05, **P < 0.01. Scale bar = 20 μm in **a**

We then examined whether aspirin treatment can inhibit the osteoclastic potential of imDCs. imDCs were induced by RANKL and M-CSF as well as aspirin (0, 50, 100, 150, or 200 μg/ml) and then stained after 7 days of culture (Fig. 3a; *P* < 0.01). The results demonstrated that aspirin suppressed the formation of OC-like cells following stimulation with RANKL, which was verified by the downregulation of OC-specific marker gene (cathepsin K (CTSK), TRAP, and calcitonin receptor (CTR)) expression in imDCs cultured under OC-inducing conditions with aspirin for 5 days (Fig. 3b; *P* < 0.01). As the reduced gene expression was most stable and significant in the 150 μg/ml aspirin group, we used this dose for treating imDCs in subsequent assays. Western blotting results showed that expression of CTSK was inhibited by aspirin treatment, which was in accordance with RT-PCR results (Fig. 3c).

### Aspirin inhibited RANKL-induced OCs by directly inhibiting the IκK/IκB/NF-Κb/NFATc1 pathway

**Fig. 4:**
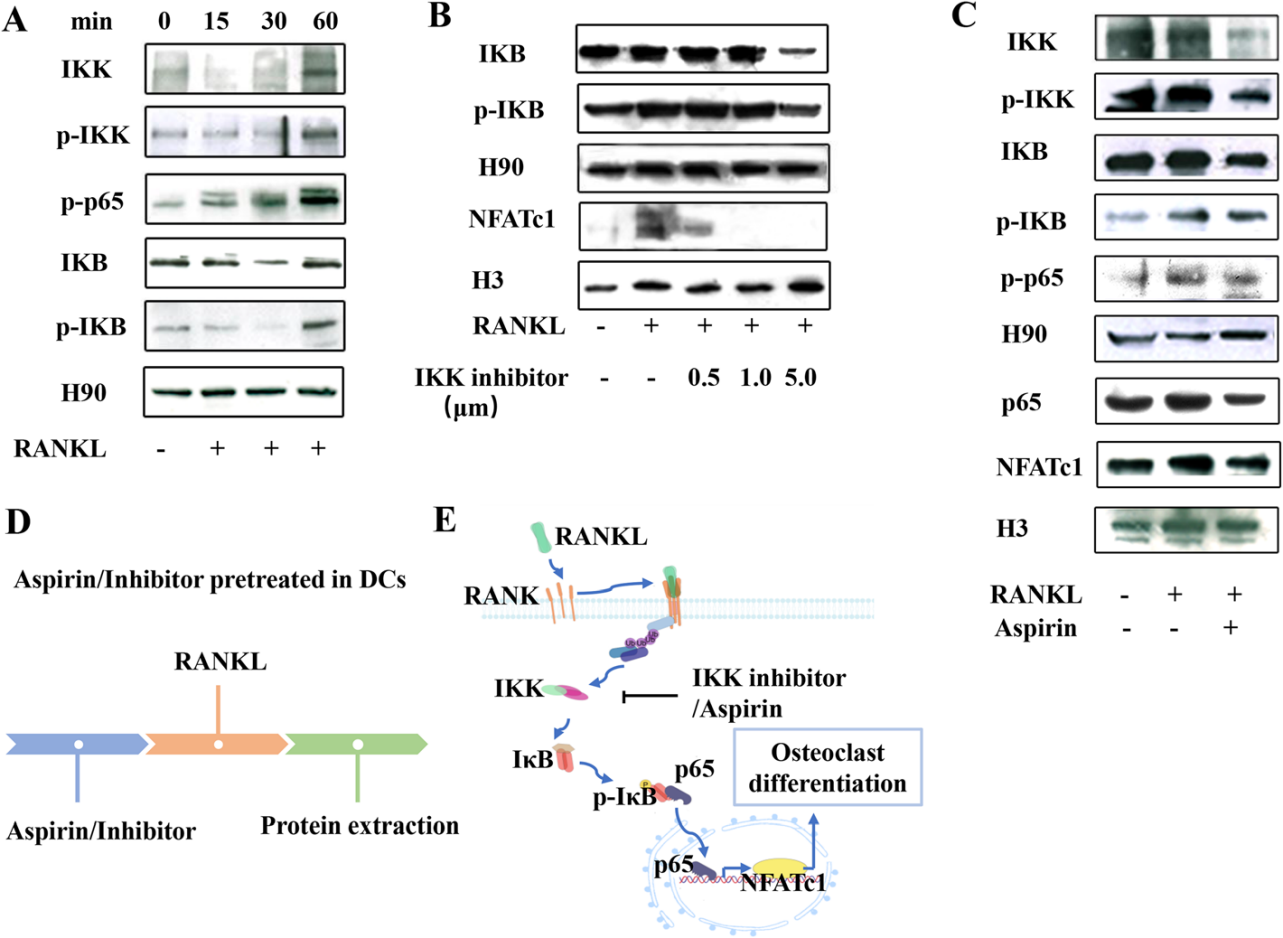
Aspirin inhibits RANKL-induced OCs via the IκK/IκB/NF-κB pathway. **a**, **b** Western blot showing that RANKL treatment upregulated IKK, p-IKK, IKB, p-IKB, and p-p65 and that IKK inhibitor treatment significantly reduced this upregulation. The expression levels of NFATc1 showed the same tendency. **c** Components of the IκK/IκB/NF-κB pathway were overexpressed after RANKL induction, which was decreased by aspirin pretreatment, with the results for NFATc1 expression being consistent. **d** Schematic representation of the timing of aspirin and IKK inhibitor treatment in our experiments. **e** Based on all of the results described above, aspirin inhibits RANKL-induced OCs via the NF-κB pathway, ultimately suppressing NFATc1 synthesis. All experiments are representative of at least three replicates

NF-κB is a dimeric transcription factor complex that plays a critical role in osteoclastogenesis [16, 17]. NFATc1 expression, which has an integral role in OC differentiation, is dependent on RANKL-NF-κB pathways. Additionally, previous studies have shown that aspirin can suppress the activity of IκB kinase (IκK)-β in the NF-κB pathway [18]. Thus, we detected the activities of NF-κB-related proteins. The results showed that IκK, p-IκK, IκB, and p-IκB expression increased after RANKL treatment and that aspirin reduced this upregulation (Fig. 4a, c). In addition, RANKL promoted P65 and NFATc1 nuclear translocation, whereas aspirin treatment suppressed this effect (Fig. 4a, c). Subsequent experiments were carried out for verification purposes. To this end, we blocked the IκK/IκB/NF-κB pathway with the IκK-β inhibitor (IMD 0354; Selleck, USA) before RANKL treatment and found that the RANKL-induced upregulation of IκB and p-IκB protein expression was suppressed. In addition, the IκK-β inhibitor blocked NFATc1 nuclear translocation (Fig. 4b).

Importantly, these results indicated that the NF-Κb/ NFATc1 pathway is involved in aspirin-mediated inhibition of the differentiation of DCs into OCs (Fig. 4d).

### Aspirin treatment showed favorable therapeutic effects on bone regeneration

**Fig. 5:**
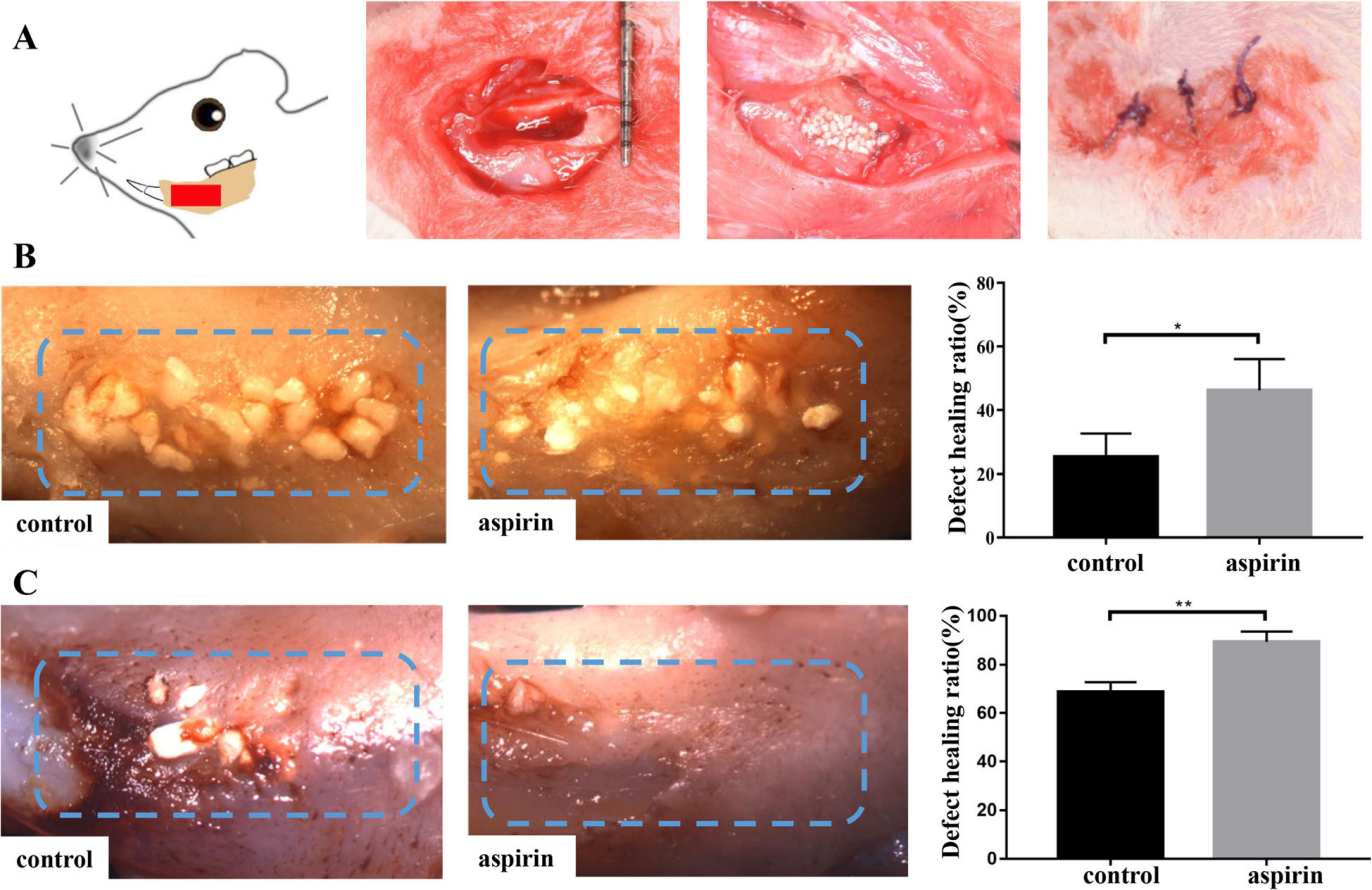
Aspirin treatment improves bone healing in the rat mandibular defect model. **a** The diagram above shows the establishment process of the alveolar bone defect model in a rat. **b** Two weeks after the surgery, the rate of bone defect healing in the aspirin group was much faster than that in the control group (*P* < 0.05). **c** Two months later, aspirin showed a clear benefit on the defect healing ratio compared to the control group (*P* < 0.01). The blue boxes in **b** and **c** are the mandibular defect area generated at the beginning of surgery. All experiments are representative of three replicates. *P < 0.05, **P < 0.01

**Fig. 6:**
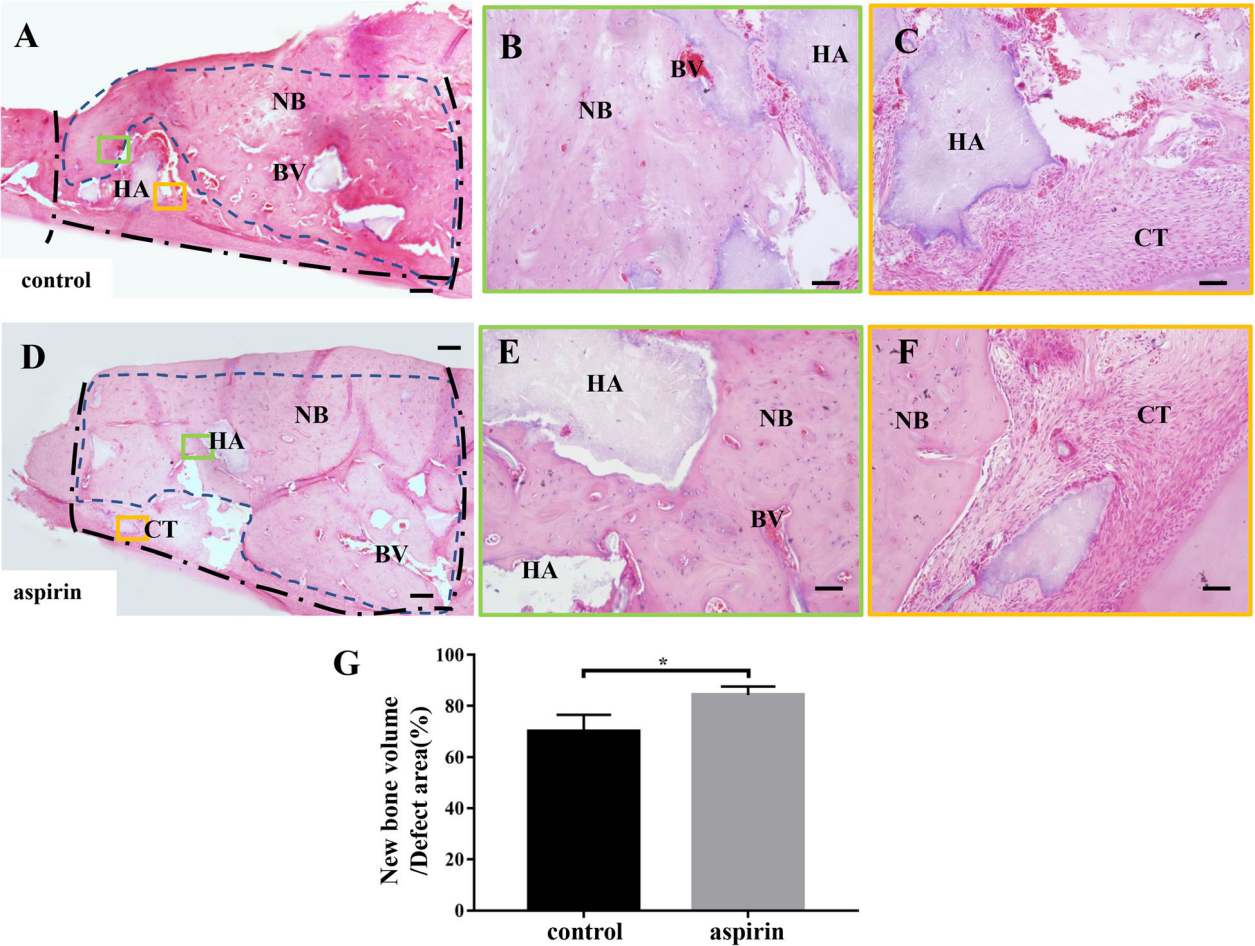
Aspirin treatment promotes HA/TCP-mediated new bone formation in the rat mandibular defect model. **a** Two months after surgery, new bone and blood vessels were observed in the control group. HA/TCP was also detected in the mineralized tissues. (Black lines show the margins of the bone defect generated during surgery. Blue lines show the area of new bone formation.) **b** The area of a green rectangle in **a** shows new bone around HA/TCP particles under a high-magnification microscope, and new blood vessels can be seen between the bone. **c** Large volumes of connective tissues remain at the edge of the bone defect, which surround the HA/TCP particles. (The area enclosed with a yellow rectangle in **a**.) **d** New bone formation almost completely repaired the bone defect in the aspirin group after 2 months. (Black lines show the margins of the bone defect generated during surgery. Blue lines show the area of new bone formation.) **e** The area in a green rectangle of **d** shows new bone formation and a large number of new surrounding blood vessels. **f** Higher magnification of the area in a yellow rectangle in **d** shows osteoblasts surrounding the surface of HA/TCP particles, with new bone formation still being active. **g** Two months later, aspirin treatment accelerated bone regeneration compared to the control group (*P* < 0.05). HA = HA/TCP particles, CT = connective tissue, BV = blood vessel, NB = new bone. Scale bar = 200 μm in **a**, **d** and **s**cale bar = 50 μm in **b**, **c**, **e**, **f**

To identify the abilities of aspirin-pretreated DDOCs in a bone defect, we generated a defect (5 mm × 2 mm × 1 mm) in the rat mandible (Fig. 5a). Defect healing in the aspirin group occurred more rapidly than in the control group at 2 weeks (Fig. 5b; *P* < 0.05). Two months later, the aspirin group showed a favorable defect-to-healing ratio (Fig. 5c; *P* < 0.01). In addition, H&E staining of bone defect sections acquired from both groups showed that aspirin promoted bone healing (Fig. 6g; P < 0.05). In the control group, new bone had formed over the bone defect surface with a moderate number of new blood vessels (Fig. 6a–c). HA/TCP particles were found in the mineralized tissue. However, numerous new bones were observed in the aspirin group, which exhibited almost complete repair of the bone defect, and new bone formation was still active around the surface of the HA/TCP particles (Fig. 6d–f).

### Aspirin inhibited OC differentiation in the bone defect model

**Fig. 7:**
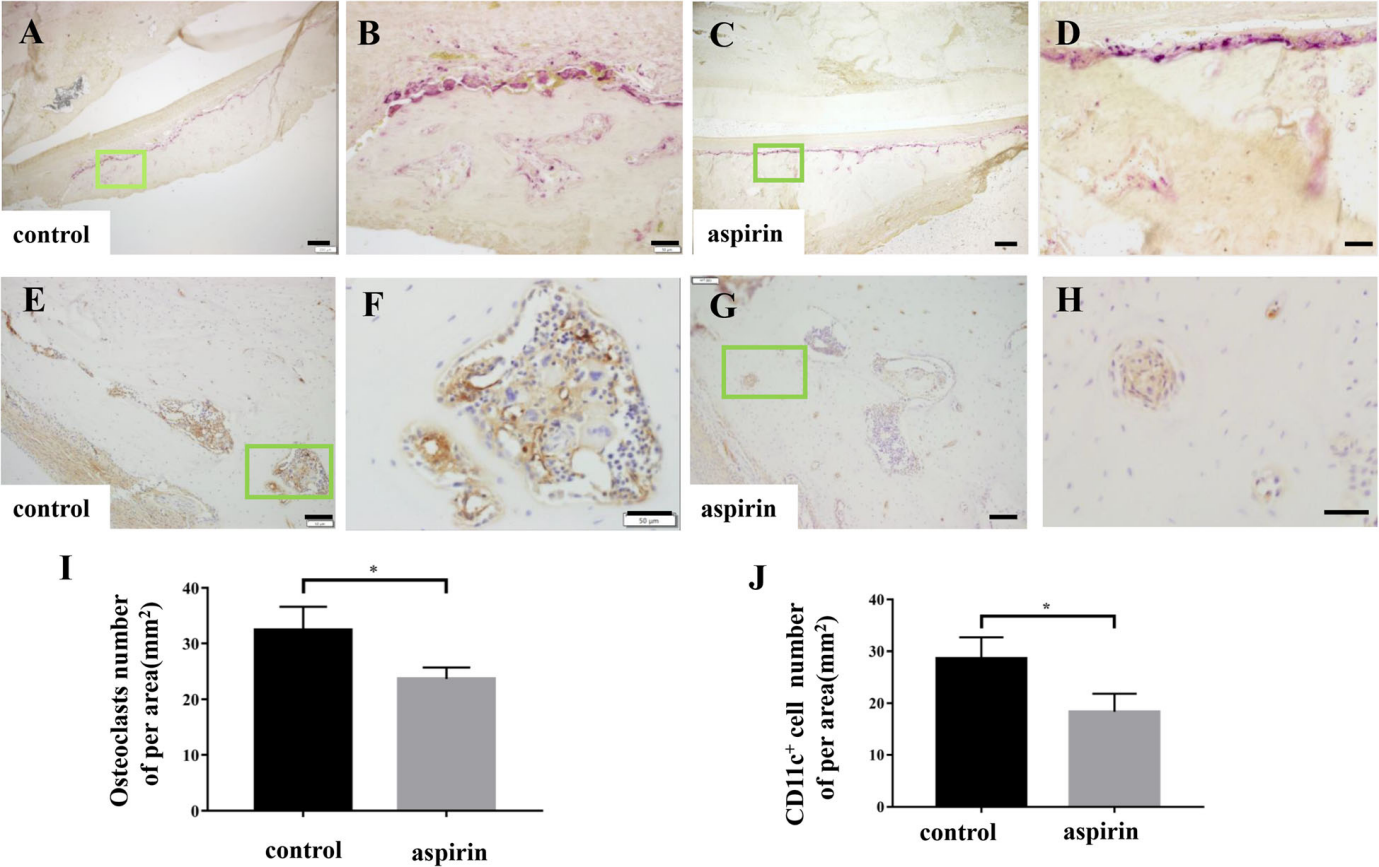
Aspirin inhibits osteoclasts during early inflammation in the rat mandibular bone defect model. **a–d** TRAP staining showed that by the 5th day after surgery, osteoclasts were increased in both the control group and aspirin group. **e–h** We performed immunohistochemical staining on sections, and the results indicated overexpression of CD11c (brown-yellow color) in both the control and aspirin groups. **i** However, compared to the control group, the aspirin group maintained lower osteoclast numbers (*P* < 0.05). **j** After surgery, CD11c^+^ cells showed a lower ratio in the aspirin group (*P* < 0.05). All experiments are representative of three replicates. *P < 0.05, **P < 0.01. Scale bar = 200 μm in **a**, **c**; scale bar = 100 μm in **e**, **g**; scale bar = 50 μm in **b**, **d**, **f**, **h**

The number of local OCs was examined in the bone defect model. According to TRAP staining, OCs remained around the bone defect in both groups showed on the third day (Fig. 7a–d). Additionally, the aspirin group exhibited a significant reduction in the number of OCs (Fig. 7i; *P* < 0.05). Most studies have indicated that immature CD11c^+^ DCs can act as OC precursors and thus become functional OCs in the inflammation-induced bone environment [19]. Thus, we adopted immunohistochemical staining to verify that the number of CD11c^+^ cells increased in both groups (Fig. 7e–h) and found a lower number of CD11c^+^ cells in the aspirin group than in the control group (Fig. 7j; *P* < 0.05).

## Discussion

Under physiological conditions, bone mass maintenance is achieved through a delicate balance between bone formation by osteoblasts and bone resorption by OCs. However, in certain inflammatory diseases, this balance is disrupted, and the activation of OCs predominates, resulting in localized bone erosion. Thus, it is important to understand the mechanisms underlying the relative activity of OCs to minimize bone injury in these inflammatory diseases [2].

Osteoclastogenesis depends on progenitor cells of the monocyte hematopoietic lineage, and these progenitor cells have been demonstrated to fuse to form OCs under the influence of M-CSF and RANKL [2, 4]. In addition, human DCs can differentiate into mature OCs [20]. In agreement with this, several researchers have generated OCs from splenic or bone marrow-derived mouse DCs in a RANKL-dependent manner [21]. Moreover, DCs are considered to be more efficient at differentiating into OCs than are monocytes in terms of fusion efficiency and the number of nuclei per OC, suggesting that DCs might have an unexpected direct role in osteoclastogenesis [22, 23]. Accordingly, downregulation of DDOC differentiation or function may be an ideal target for the treatment of pathological bone diseases.

Aspirin is an NSAID commonly used in clinical applications because of its various antipyretic, analgesic, antirheumatic, and anti-platelet aggregation effects [19, 24]. The properties of aspirin involve a variety of pathways, such as inhibition of COX-2 and COX-1 and prostaglandin E2 (PGE2) activities [7, 25, 26]. PGE2 is a multifunctional regulator of bone metabolism that affects bone resorption and formation [27], and previous studies have shown that treatment with COX-2-specific NSAIDS reduce levels of PGE2 while negatively affecting bone healing [28]. The inhibitory effect of aspirin on bone healing has also been demonstrated in four animal studies and a human in vitro study [8, 29–32]. Nonetheless, a recent study indicated that aspirin has positive effects on cranial bone regeneration in miniature pigs, partly by promoting bone marrow mesenchymal stem cell-based osteogenesis [8]. Moreover, epidemiological studies have demonstrated that frequent use of aspirin may have a moderate beneficial effect on bone mineral density in postmenopausal women [33]. In general, the controversial results may depend on the preparations of the drug, modes of administration, doses, and ability to inhibit COX-2. Aspirin inhibits OCs [34] by promoting telomerase activity and also dramatically activates osteoblasts [26, 35]. In addition, exogenous PGE2 may strongly stimulate bone resorption in bone organ cultures and osteoclast differentiation in bone marrow cultures. PGE2 has biphasic effects on RANKL-induced OC formation in bone marrow cultures [27]. For example, PGE2 increases RANKL-induced OC differentiation in RAW264.7 cells [36] but inhibits differentiation in cultured human peripheral blood mononuclear cells (PBMCs) [37]. Furthermore, aspirin was shown to inhibit RANKL-induced osteoclastogenesis in RAW264.7 cells [14] and to decrease bone resorption by downregulating PGE2 both in vivo and in vitro [38]. Regardless, the inhibitory potential and mechanisms of aspirin with regard to the differentiation of DCs into OCs have not been elucidated.

In this study, we observed that DCs were able to induce TRAP^+^ OC formation in response to RANKL and that this process was inhibited by aspirin. We further investigated the underlying molecular mechanisms involving aspirin in OC differentiation and function. The RANKL/RANK/osteoprotegerin (OPG) axis is the classic regulatory system of bone metabolism, and it can regulate OC differentiation [39, 40]. Binding of RANKL to its receptor RANK results in rapid recruitment of multiple intracellular signaling molecules, such as MAPK, NF-κB, AP-1, TRAFs, and NFATc1, with NF-κB being the most important factor [41]. Indeed, NF-κB signaling has been shown to play an essential role in osteoclastogenesis, and its suppression can inhibit NFATc1 expression [42]. Previous research demonstrated that the NF-κB inhibitor (−)-dehydroxymethylepoxyquinomicin suppresses RANKL-induced osteoclastogenesis by downregulating NFATc1 expression [43]. In the present study, we found that treatment with aspirin suppressed RANKL-induced NF-κB signaling pathways.

Multiple previous studies have established that during osteoclastogenesis, NFATc1 is the specific transcription factor that regulates OC-specific genes, such as cathepsin K, TRAP, and CTR, involved in the regulation of RANKL-mediated OC differentiation, fusion, and activation [39, 44]. The present data suggest that stimulation of imDCs with RANKL leads to expression of NFATc1 and that aspirin treatment reduces RANKL-induced NFATc1 activation. Collectively, these results indicate that aspirin effectively inhibits OC differentiation in imDCs by regulating NF-κB induction and NFATc1 following RANKL/RANK interaction.

## Conclusion

In summary, our results demonstrate that aspirin downregulates the RANKL-mediated induction of NF-κB and NFATc1. This effect then participates in downregulating expression of the downstream nuclear transcription factor NFATc1 and inhibiting RANKL-induced OC differentiation in preosteoclastic imDCs. However, further investigations into the role of imDCs in bone resorption and the feasibility of using aspirin in clinical applications are still required.

## Data Availability

All data analyzed during the current study are included in this published article.

## Abbreviations

α-MEM: α-minimum essential medium
BMSC: Bone marrow mesenchymal stem cell
CTR: Calcitonin receptor
CFSE: Carboxyfluorescein succinimidyl ester
CTSK: Cathepsin K
COX-1: Cyclooxygenase-1
COX-2: Cyclooxygenase-2
DCs: Dendritic cells
DDOCs: Dendritic cell-derived osteoclasts
H&E: Hematoxylin and eosin
imDCs: Immature dendritic cells
M-CSF: Macrophage colony-stimulating factor
MLR: Mixed lymphocyte reaction
mAbs: Monoclonal antibodies
NSAID: Nonsteroidal anti-inflammatory drug
NF-κB: Nuclear factor kappa B
NFATc1: Nuclear factor of activated T cell, cytoplasmic 1
OC: Osteoclast
OPG: Osteoprotegerin
PFA: Paraformaldehyde
PBMC: Peripheral blood mononuclear cell
PGE_2_: Prostaglandin E2
RANKL: Receptor activator of nuclear factor-κB ligand
RT-PCR: Reverse transcription polymerase chain reaction
TRAP: Tartrate-resistant acid phosphatase
